# Supplementary material for: Early Childhood Stimulating Environment Predicts Later Childhood Resilience in an Indian Longitudinal Birth Cohort Study
Source: Children (Basel). 2022 Nov 9;9(11):1721. doi: 10.3390/children9111721 (PMC9689234; doi:10.3390/children9111721)
Supplement: Supplementary file 1 [file children-09-01721-s001.zip › children-1973057-supplementary.pdf]

Supplementary Table S1: Distribution of various domains of resilience scores at 9 years of age in children of the MAL-ED cohort (*n*=205)

	**Mean (SD) scores**	**Median (IQR) scores**
Individual domain	26.34 (3.19)	27 (25-29)
Caregiving domain	19.52 (1.64)	20 (19 - 21)
Contextual domain	20 (1.80)	20 (19 - 21)
Total resilience scores	65.86 (4.69)	67 (63 - 69)

MAL-ED - The 'Etiology, Risk Factors and Interactions of Enteric Infections and Malnutrition and the Consequences for Child Health and Development' Study, IQR – Interquartile range
